# Effectiveness of Cardiac Rehabilitation in Enhancing Clinical Outcomes for Coronary Artery Disease: A Systematic Analysis

**DOI:** 10.7759/cureus.69224

**Published:** 2024-09-11

**Authors:** Pravallika Myneni, Monicaa Bodduluri, Sai T Gadde, Rithish Nimmagadda, Male Manvitha, Sindhu Chowdary Valiveti, Sweta Sahu, Salma Younas

**Affiliations:** 1 General Medicine, Katuri Medical College and Hospital, Guntur, IND; 2 Internal Medicine, Katuri Medical College and Hospital, Guntur, IND; 3 General Medicine, All India Institute of Medical Sciences (AIIMS) Mangalagiri, Mangalagiri, IND; 4 Internal Medicine, California Institute of Behavioral Neurosciences & Psychology, Fairfield, USA; 5 Internal Medicine, Sri Venkateswara Institute of Medical Sciences (SVIMS) Sri Padmavathi Medical College for Women (SPMCW), Tirupati, IND; 6 General Medicine, Sri Venkateswara Institute of Medical Sciences (SVIMS) Sri Padmavathi Medical College for Women (SPMCW), Tirupati, IND; 7 Internal Medicine, JJM Medical College, Davanagere, IND; 8 Pharmacy, Punjab University College of Pharmacy, Lahore, PAK

**Keywords:** cardiac rehabilitation, coronary artery disease, coronary plaque volume, exercise, exercise tolerance, high-intensity interval training

## Abstract

Cardiac rehabilitation (CR) is a structured intervention aimed at improving the clinical outcomes for patients with coronary artery disease (CAD). This systematic review assesses how well different types of CR, such as high-intensity interval training (HIIT), moderate-intensity continuous training (MICT), Nordic walking (NW), and home-based cardiac rehabilitation (HBCR), improve exercise capacity, quality of life, and lower death and illness rates. The objective is to assess the effectiveness of cardiovascular rehabilitation programs in enhancing clinical outcomes for patients diagnosed with CAD. A comprehensive literature search was conducted using the Preferred Reporting Items for Systematic Reviews and Meta-Analyses (PRISMA) model across Google Scholar, EMBASE, PubMed, Medline, and web browsers. Keywords such as "cardiac rehabilitation," "coronary artery disease," "exercise testing," "VO2 peak," and "physical activity" were used in different combinations. Studies were included if they were randomized controlled trials, observational studies, or longitudinal studies published after 2013 in English, with a focus on the impact of CR on CAD. Articles were excluded if they were reviews, meta-analyses, or did not meet the keyword requirements.

A total of 375 articles were initially identified with relevant citations. After further screening, 10 studies met the inclusion criteria for analysis. The studies reviewed demonstrated that all forms of CR, including HIIT, MICT, NW, and HBCR, significantly improved exercise capacity and quality of life, and reduced depression severity among CAD patients. Nordic walking showed marked improvements in functional capacity, while HIIT resulted in higher VO2 peak levels compared to moderate-intensity exercise. Home-based CR showed greater adherence rates, especially among older patients and those with strong family support. The results also highlighted the importance of individualized exercise programs to enhance adherence and outcomes. Cardiac rehabilitation is a vital component of secondary prevention in CAD patients, significantly improving clinical outcomes, including exercise capacity, quality of life, and mortality rates. The findings underscore the importance of maintaining and expanding access to CR programs and tailoring interventions to patient needs to optimize long-term health outcomes. Future research should explore the comparative effectiveness of different CR modalities and strategies to increase patient adherence.

## Introduction and background

Coronary artery disease (CAD) is a condition where the coronary arteries, which supply blood to the heart muscle, become narrowed or blocked due to the buildup of plaque (atherosclerosis). This can lead to chest pain (angina), shortness of breath, or heart attacks if the blood flow is significantly reduced or blocked [1]. Cardiac rehabilitation (CR) is a multidisciplinary intervention aimed at improving the health outcomes of patients with CAD by enhancing physical capacity, quality of life, and psychological well-being. CR enhances heart function, reduces symptoms, and decreases the risk of future cardiac events by improving physical fitness, lowering blood pressure, and optimizing lipid levels. CR programs typically encompass various components, including exercise training, health education, cardiovascular risk management, and psychological support, which are customized to meet the specific needs of individual patients [2]. As a secondary prevention strategy, modern CR not only targets cardiovascular risk reduction but also emphasizes promoting patient well-being and improving health-related quality of life (HRQoL) [3].

CAD remains the leading cause of death globally, accounting for approximately 17.9 million deaths annually, which represents about 32% of all global deaths according to the World Health Organization (WHO) [4]. In high-income countries, CAD is responsible for one in every five deaths [5]. In the United States alone, CAD affects an estimated 18.2 million adults aged 20 years and older, which is approximately 6.7% of the population [6]. 

CR is considered a cost-effective approach for managing coronary heart disease (CHD), providing significant benefits by enhancing functional capacity, reducing acute hospital admissions, and improving overall patient outcomes [7]. The primary objectives of CR are to increase daily energy expenditure, improve exercise tolerance, and lower the risk of cardiovascular mortality and hospitalizations. Participating in CR has been shown to improve cardiovascular mortality, reduce the risk of hospitalizations, enhance fitness levels, and improve HRQoL in patients with CAD and heart failure (HF) [8].

Coronary artery disease is a leading cause of mortality and morbidity globally, particularly in developed countries. Despite the advancements in coronary rejuvenation techniques, such as percutaneous coronary intervention (PCI) and coronary artery bypass graft surgery (CABG), which have improved survival rates in CAD patients post-myocardial infarction (MI), many patients still experience diminished functional capacity, a high prevalence of depression, and a markedly poor quality of life [9]. The benefits of standard CR, which includes moderate-to-vigorous intensity continuous exercise, in improving exercise capacity, mental well-being, and quality of life are well-documented [10].

Recent evidence from a meta-analysis involving over 10,000 patients across 47 randomized studies has demonstrated that CR significantly reduces both cardiac and all-cause mortality, highlighting its role as an essential intervention for secondary prevention in patients with CAD [11].

One of the key components of CR is exercise training (ET), which has been demonstrated to increase exercise tolerance, lower cholesterol levels, alleviate symptoms, enhance psychosocial well-being, and reduce mortality [12]. ET serves as a critical primary treatment and secondary prevention strategy for individuals with CAD. However, despite the established benefits of formal CR programs, participation remains relatively low. Barriers such as geographical distance, limited access to healthcare facilities, and other logistical challenges contribute to low participation rates in hospital-based CR programs [13]. In response to these challenges, home-based CR has emerged as a viable alternative, with several studies revealing that it is equally effective and safe as hospital-based programs [14].

While home-based CR offers convenience and flexibility, adherence to these programs is variable. Some studies suggest that patients may favor home-based rehabilitation due to its accessibility and reduced logistical burden. However, the adherence rates to home-based CR are debated. For example, McKelvie et al. [15] reported lower compliance in the home-based group compared to those in supervised training, while Jolly et al. [16] found higher adherence rates among patients participating in home-based CR programs. This discrepancy may be attributed to different methodologies employed in home-based CR programs and regional variations in healthcare delivery. In contrast to center-based CR, patients assigned to home-based CR reported a broader range of reasons for non-adherence, highlighting the complexity of factors influencing patient compliance [17].

Fitness is recognized as a strong predictive indicator for future cardiovascular events and is independently associated with enhanced quality of life in cardiac patients [18]. The improvements in cardiorespiratory fitness observed in patients after rehabilitation are closely linked with reduced mortality and morbidity rates [19-21]. Despite the well-established therapeutic significance of fitness in cardiac patients, there have been limited attempts to synthesize the existing research comprehensively [22]. Therefore, a systematic examination of the available evidence is crucial to understanding the broader implications of CR and its role in improving clinical outcomes.

Short-term, specific goals set by patients or in collaboration with healthcare professionals tend to be more effective in achieving desired outcomes [23-27]. Self-monitoring, facilitated by tools like diaries or activity records, helps patients maintain awareness of their current behavior and make necessary adjustments to meet their goals [28]. Regular follow-ups and interactions with healthcare professionals further support goal attainment and foster long-term adherence to healthy lifestyle changes [29-32].

CR programs are thus designed to offer a holistic approach to patient care, addressing not only the physical but also the psychological and social dimensions of recovery. The comprehensive nature of these programs helps to bridge the gap between hospital-based care and the patient's return to daily life, ensuring continuity of care and sustained health improvements.

However, despite these benefits, gaps remain in the literature regarding the comparative effectiveness of different CR modalities. For instance, high-intensity interval training (HIIT) and moderate-intensity continuous training (MICT) have both been utilized in CR, but there is a need for further studies to evaluate their relative efficacy in diverse patient populations. Similarly, while home-based programs have gained acceptance as a practical alternative to center-based CR, more research is needed to understand the factors influencing adherence and long-term outcomes in these settings.

This systematic review aims to assess the effectiveness of various CR modalities, including HIIT, MICT, and home-based programs, in improving clinical outcomes such as exercise capacity, quality of life, and mortality rates in patients with CAD. By synthesizing current evidence, this review seeks to provide a comprehensive understanding of the impact of different CR approaches on patient health and to identify gaps for future research.

## Review

Study design

This systematic review follows the Preferred Reporting Items for Systematic Reviews and Meta-Analyses (PRISMA) guidelines to evaluate the effectiveness of CR in enhancing clinical outcomes for patients with CAD. The review includes studies published in English from 2013 to 2023, focusing on the impact of different CR modalities on exercise capacity, quality of life, and functional outcomes.

Search strategy

A comprehensive literature search was conducted across several electronic databases, including PubMed, EMBASE, Medline, and Google Scholar. The search was carried out from January 1, 2013, to December 31, 2023. Specific search terms used included "cardiac rehabilitation," "coronary artery disease," "exercise capacity," "physical activity," "high-intensity interval training," and "moderate-intensity continuous training." Boolean operators ("AND," "OR") were employed to refine search results, for example, "Cardiac rehabilitation AND coronary artery disease" and "Exercise capacity OR physical activity." The initial search yielded 375 records.

Inclusion and exclusion criteria

To simplify the process of choosing the articles to include in our evaluation, a list of factors on which articles are included and excluded is mentioned. The list seeks to choose the pertinent publications that will serve our literature review's objective (Table 1).

**Table 1:** Inclusion and exclusion criteria

Inclusion criteria	Exclusion criteria
Observational studies	Reviews and met analysis
Articles addressing the topic	Articles not meeting keywords
Articles published after 2013	Articles published before 2013
English language	Abstract only publications
Full-text article	No available digital copies of articles
Randomized clinical trials	Language other than English

Data extraction

Data extraction was conducted independently by two reviewers utilizing a standardized data extraction form to ensure consistency and accuracy. Key information extracted from each study included study characteristics, such as the author, year, country, and study design. Participant characteristics were also recorded, including sample size. Details of the cardiac rehabilitation interventions were meticulously documented, encompassing the type, duration, and intensity of the interventions. The primary outcome measures extracted focused on exercise capacity, quality of life, and functional outcomes. The risk of bias for each study was also assessed, providing a comprehensive overview of the robustness of the included studies.

Bias assessment

Discrepancies in data extraction were resolved by consensus or by consultation with a third reviewer. The Mixed Methods Appraisal Tool (MMAT), which allows for the evaluation of qualitative and quantitative, is used for mixed methods studies. Prior mixed method systematic reviews have used and validated it. Each study was evaluated independently by two assessors, with disagreements settled through discussion. There were quality ratings given, ranging from 0% to 100%, but no research was disqualified due to poor quality. For quantitative studies, the Cochrane tool is used for the assessment of bias risk.

Results

**Figure 1:**
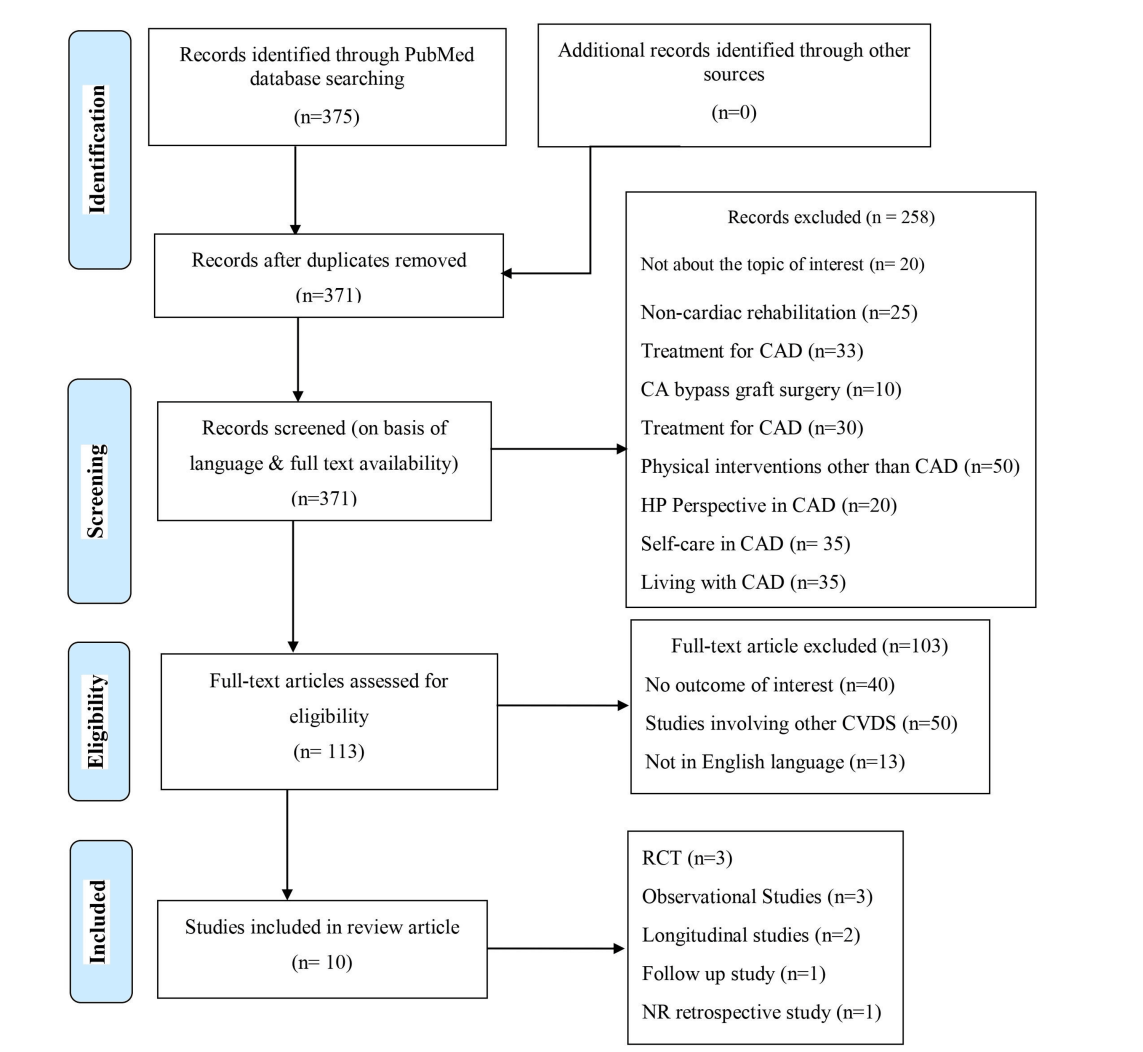
PRISMA flow diagram PRISMA: Preferred Reporting Items for Systematic Reviews and Meta-Analyses, CAD: Coronary Artery Disease, CA bypass graft surgery: Coronary Artery Bypass Graft Surgery, HP Perspective: Health Professional Perspective, RCT: Randomized Controlled Trial, CVDs: Cardiovascular Diseases, NR retrospective study: Non-Randomized Retrospective Study

The results are tabulated in the following table (Table 2). This study consists of three randomized control trials, one longitudinal randomized study, three observational studies, one longitudinal observational study, one-year follow-up of cardiac rehabilitation, and one non-randomized retrospective study. A total of 10 studies are included in this study (Figure 1).

**Table 2:** Study table including the article with analysis of each study using Mixed Methods Appraisal Tool (MMAT) and Cochrane tool HIIT: high-intensity interval training, MICT: moderate-intensity continuous training, QoL: quality of life, CR: cardiac rehabilitation, CAD: coronary artery disease, PAD: peripheral artery disease, CRF: cardiorespiratory fitness, PCI: percutaneous coronary intervention, DM: diabetes mellitus, MET: metabolic equivalent of task

Authors	Study design	Cardiac Rehabilitation Program	Patient Sample	Clinical Measurements	Key findings	Bias Score	Study type
Reed et al., 2021 [33]	Randomized Clinical Trial	High-intensity cardiac training, Nordic walking (NW), and moderate-intensity cardiac training	135	Improving Functional Capacity Additionally, the effects on brain-derived neurotrophic factor (BDNF), QoL, and depression severity were also measured	When compared to HIIT and MICT, a considerably larger gain in functional capacity was attained after NW. The severity of depression decreased and QoL improved.	100%	(Both qualitative and quantitative)
Pinto et al., 2021 [34]	Longitudinal Randomized Study	Periodization of long-term exercise	50	Components of physical fitness that are associated with health, such as endurance of muscles, functionality of skeletal muscles, and composition of the body.	This research will help develop evidence-based exercise prescription strategies to continue exercising after hospital-based CR programs have ended.	75%	Both (qualitative and quantitative studies)
Bruno et al., 2022 [35]	One-year follow-up of cardiac rehabilitation	A medical education program, at least 5-6 workout sessions, involving resistance, continuous or interval training, and 7 respiratory training were all included in the cardiac rehabilitation program.	259	Observe how the CRF level changes in CAD patients before, throughout, and after their CR program.	The CRF improvement was just below the 1.5 METs reported as the average value in other studies.	50%	Both (qualitative and quantitative studies)
Nguyen et al., 2021 [36]	Observational study	Walking for 30-45 minutes	164	Examine changes in VO2peak between individuals with PAD and CAD after 6 months of cardiac rehabilitation.	Patients with CAD experienced larger VO2peak improvements than those with PAD. Inclusion of PAD subjects in Cardiac Rehab because of improvements in VO2 peak.	Low risk of bias	Quantitative studies
Nilsson et al., 2018 [37]	Observational study	On a treadmill, two described protocols were created. 1) walking protocol 2) Running protocol	133	Effect 15 months after CR entrance on VO2peak. Health-related quality of life was measured.	Improved and preserved the quality of life and VO2 peak	Low risk of bias	Quantitative studies
Guimarães et al., 2023 [38]	Observational study	CR program was divided into three groups Aqua Walk Treadmill walk Non-exercise control group	60	Analyze the impact of aqua walking (AW) versus conventional over-ground walking (CON) on cardiorespiratory fitness and CAD in older persons with osteoarthritis in the lower limbs.	Aqua walk appears to be an achievable choice for over-ground walking as an exercise paradigm for cardiac rehabilitation in older people with CAD and osteoarthritis.	Low risk of bias	Quantitative studies
Yokoyama et al., 2019 [39]	Randomized control trial	Exercise and physical training	32	The effects of CR incorporating vigorous exercise (PA) on the volume and elements of coronary plaque in people with acute coronary syndrome (ACS).	The intense CR and standard CR groups showed no discernible differences in PV or components	Low risk of bias	Quantitative Method
Hu et al., 2020 [40]	Non-randomized retrospective study	Evaluation of normal care, exercise alone, and cardiac rehabilitation (exercise + education) for patients with coronary artery disease	492	Functional walking ability, risk factor management, and morbidities in cardiac rehabilitation follow-up	Program for cardiac rehabilitation (exercise + education) compared to individuals with coronary artery disease who merely exercise and receive standard therapy, reduced morbidities, controlled risk variables, and enhanced functional walking capacity were noted.	75%	Both (qualitative and quantitative studies)
Navarro et al., 2017 [41]	Longitudinal observational study	Exercise training	700	1) To assess the effect of CR on cardiovascular events and death in DM patients following PCI. and (2) to contrast how CR affects the aforementioned results relative to patients with and without DM.	The mortality rate is mitigated in patients with DM who underwent percutaneous coronary artery intervention.	50%	Both (qualitative and quantitative studies)
Okur et al., 2022 [42]	Randomized control trial	Two HIIT programs are short and long. High-intensity interval training (HIIT) programs and moderate-intensity continuous training (MICT)	20	Functional capacity and quality of life of patients	In terms of increasing the maximal exercise capacity of CAD patients, HIIT programs outperformed MICT, and all three programs had a comparable impact on quality of life.	50%	Both (qualitative and quantitative studies)

The studies included in this review collectively provide evidence of the effectiveness of various CR programs for patients with CAD and related conditions. However, the range of sample sizes and the quality assessment scores varied considerably across the studies, affecting the strength of the conclusions drawn.

The quality scores ranged from 50% to 100%, with some studies, such as those by Reed et al. (2021) and Hu et al. (2020), demonstrating high methodological quality (100% and 75% respectively) [33,40]. In contrast, other studies, like those by Bruno et al. (2022) and Navarro et al. (2017), scored lower (50%) [35,42]. This variation suggests that while some findings are robust, others may be more susceptible to bias or methodological limitations, such as small sample sizes or less rigorous study designs.

HIIT often outperforms MICT in improving exercise capacity, as noted in studies by Reed et al. (2021) and Okur et al. (2022) [33,42]. Potential confounding factors, such as differences in patient characteristics (e.g., baseline health status, age, or comorbidities), could influence the outcomes. The impact of intervention duration, adherence rates, and variations in the delivery of HIIT and MICT programs across studies could affect the comparability of results. The studies also varied in their sample sizes, ranging from small groups (e.g., 20 participants in Okur et al., 2022) to larger cohorts (e.g., 700 participants in Navarro et al., 2017) [41,42]. Smaller sample sizes may limit the statistical power of the findings and increase the risk of Type II errors.

For instance, studies like Guimarães et al. (2023) provide evidence for the feasibility of aqua walking for older patients with CAD and osteoarthritis [38]. However, the practical application of such findings may depend on patient-specific factors, such as mobility issues or access to appropriate facilities.

Discussion

CR has consistently shown benefits in improving clinical outcomes for patients with CAD. This review included studies of various CR modalities-such as HIIT, MICT, Nordic walking (NW), home-based cardiac rehabilitation (HBCR), and water-based exercises-to evaluate their impact on exercise capacity, quality of life, morbidity, and mortality.

The comparison between HIIT and MICT suggests that HIIT may provide superior improvements in cardiorespiratory fitness (CRF) and VO2 peak, key prognostic markers for mortality in CAD patients. Okur et al. demonstrated that HIIT significantly enhances functional capacity, with greater improvements observed in VO2 peak compared to MICT. This finding is consistent with a broad body of evidence suggesting that HIIT's variable intensity better stimulates cardiovascular adaptations, leading to greater gains in fitness. However, this benefit must be weighed against the potential risks and challenges associated with HIIT, including the need for careful patient monitoring, especially in those with advanced disease or comorbidities [42].

The randomized controlled trial (RCT) by Reed et al. [33] demonstrated that NW significantly improves functional capacity, a key predictor of future cardiovascular events [38], in CAD patients compared to HIIT and MICT. All exercise types reduced depression severity and enhanced quality of life, showing substantial positive effects on functional capacity. CR programs can confidently use NW to improve physical and mental health for patients post-coronary revascularization, depending on available resources, staff expertise, and patient preferences.

ET is recognized as a beneficial adjunct in managing chronic cardiac diseases, with well-established benefits such as increased exercise tolerance and improved quality of life [43]. However, studies often report reductions in long-term exercise ability, high dropout rates, and low adherence to physical activity recommendations after short-term (up to three months) CR programs [44-46]. To enhance outcomes and adherence, ET programs should be closely monitored and tailored to individual needs [45]. Several studies in the review addressed the effectiveness of different CR components and highlighted the importance of individualized exercise prescriptions. For example, the research by Bruno et al. reported that improvements in CRF were somewhat lower than expected in outpatients, likely due to differences in exercise intensity and duration. The findings emphasize the need for careful patient assessment and tailored interventions that consider baseline fitness levels and specific cardiovascular risk profiles [35].

Yokoyama et al. [39] conducted a single-blinded clinical trial to assess the impact of HBCR on health and quality of life, noting reduced emergency hospital visits. While both center-based cardiac rehabilitation (CBCR) and HBCR show similar effects on risk factors, quality of life, morbidity, and mortality, adherence was higher in HBCR, particularly among patients over 65 and those with strong family support [47,48]. HBCR programs typically include exercise, cardiovascular risk reduction, smoking cessation, dietary advice, psychological support, and medication compliance [45]. Improved CRF, a critical mortality predictor [49], is achieved through ET, with an 8-35% reduction in mortality risk per increase in metabolic equivalent of task (MET) [43].

HIIT is found to produce greater improvements in peak oxygen uptake (VO2 peak) in patients with peripheral artery disease (PAD) and CAD compared to moderate-intensity exercise [47]. Future research should consider patient enjoyment to ensure sustained exercise adherence. After initial barriers to walking are overcome, HIIT could be gradually introduced [48]. The American Heart Association emphasizes the importance of measuring CRF as a practical tool for enhancing patient management and outcomes [50]. Guimarães et al. [38] reported body composition changes with water-based exercise therapy, though the evidence is limited, and some findings are inconsistent [51-54].

CR programs for CAD patients post-myocardial infarction, percutaneous coronary intervention, or coronary artery bypass grafting are associated with improved shuttle walk test results, cardiovascular knowledge, dietary and exercise behaviors, and reductions in body mass index and depressive symptoms [40]. Although studies highlight the positive effects of CR on quality of life, weight management, exercise tolerance, and risk factor control in diabetes mellitus (DM) patients, there is limited evidence on its impact on mortality [55-57]. Armstrong et al. [58] found that CR significantly reduces cardiac hospitalizations and mortality in DM patients, with outcomes comparable to non-DM patients.

Study limitations

While the evidence supports the use of various CR modalities, the review also reveals notable limitations that must be addressed to strengthen the validity and generalizability of the findings. A significant limitation across the included studies is the heterogeneity in study design, sample sizes, patient populations, and intervention types. For instance, the duration of CR programs ranged from short-term interventions (e.g., three-month programs) to more extended follow-up studies, leading to variability in reported outcomes such as exercise adherence, morbidity, and mortality rates.

The quality assessment scores of the studies included in the review varied widely, from 50% to 100%, reflecting differences in methodological rigor. Some studies did not account for key confounding factors, such as baseline comorbidities, demographic differences, or variations in adherence, which could significantly impact the outcomes. For example, variations in patient characteristics (e.g., age, baseline fitness level, socioeconomic status) or CR delivery (e.g., home-based vs. center-based) could contribute to the observed differences in outcomes.

Future recommendations

CR is important in improving clinical outcomes in CAD patients but also underscores the need for a more nuanced understanding of how different CR modalities can be tailored to meet individual patient needs. Diversity of CR approaches and their varying impacts on outcomes, clinicians should consider a patient-centered approach that incorporates multiple modalities, patient preferences, and logistical constraints. This could help maximize the benefits of CR and improve patient adherence and satisfaction.

Future studies should focus on larger, well-designed randomized controlled trials that standardize CR protocols and include diverse patient populations. There is a need for more research on the long-term effects of different CR modalities and their impact on quality of life, adherence, and cardiovascular health. Understanding these factors will be crucial for developing evidence-based guidelines and optimizing CR delivery in clinical practice.

## Conclusions

CR is a critical component of secondary prevention for CAD and is widely recommended in global clinical guidelines. The findings from this review demonstrate that CR, particularly when personalized to patient needs, significantly improves clinical outcomes, including reduced cardiovascular mortality, decreased hospital readmissions, and enhanced quality of life. While studies show that CR does not significantly alter coronary plaque volume, it offers substantial benefits for patients with comorbidities such as diabetes mellitus by improving functional capacity and reducing mortality. Given these positive outcomes, it is essential for clinicians to prioritize the integration of individualized CR programs into standard care for CAD patients. Future research should focus on understanding the long-term effects of various CR modalities and determining the most effective approaches for different patient subgroups. Additionally, efforts should be made to expand access to CR programs, particularly for underserved populations, to ensure that all patients with CAD can benefit from these life-saving interventions. Maintaining and increasing funding for these programs is crucial to enhancing their reach and impact, ultimately improving long-term health outcomes for patients with CAD.
